# The *agr* quorum sensing system in *Staphylococcus aureus* cells mediates death of sub-population

**DOI:** 10.1186/s13104-018-3600-6

**Published:** 2018-07-24

**Authors:** Wilhelm Paulander, Anders Nissen Varming, Martin Saxtorph Bojer, Cathrine Friberg, Kristoffer Bæk, Hanne Ingmer

**Affiliations:** 1 Department of Veterinary and Animal Sciences, University of Copenhagen, Frederiksberg, Denmark; 2 Present Address: ALK-Abelló, Horsholm, Denmark; 3 Present Address: Novo Nordisk, Gentofte, Denmark; 4 Imperial College London, London, UK

**Keywords:** *S. aureus*, *agr*, Quorum sensing, Cell death, Autolysis, Competition, Fitness

## Abstract

**Objective:**

In the human pathogen, *Staphylococcus aureus*, the *agr* quorum sensing system controls expression of a multitude of virulence factors and yet, *agr* negative cells frequently arise both in the laboratory and in some infections. The aim of this study was to examine the possible reasons behind this phenomenon.

**Results:**

We examined viability of wild type and *agr* mutant cell cultures using a live-dead stain and observed that in stationary phase, 3% of the wild type population became non-viable whereas for *agr* mutant cells non-viable cells were barely detectable. The effect appears to be mediated by RNAIII, the effector molecule of *agr*, as ectopic overexpression of RNAIII resulted in 60% of the population becoming non-viable. This effect was not due to toxicity from delta toxin that is encoded by the *hld* gene located within RNAIII as *hld* overexpression did not cause cell death. Importantly, lysed *S. aureus* cells promoted bacterial growth. Our data suggest that RNAIII mediated cell death of *agr* positive but not *agr* negative cells provides a selective advantage to the *agr* negative cell population and may contribute to the common appearance of *agr* negative cells in *S. aureus* populations.

**Electronic supplementary material:**

The online version of this article (10.1186/s13104-018-3600-6) contains supplementary material, which is available to authorized users.

## Introduction

*Staphylococcus aureus* is an opportunistic human pathogen that can cause a variety of infections [1]. The expression of virulence factors is to a large extent controlled by the *agr* quorum sensing (QS) system composed of the response regulator, AgrA and the sensor histidine kinase, AgrC that in response to auto-inducing peptides expresses a regulatory RNA, RNAIII. At high cell density RNAIII mediates the transition from production of host matrix binding and immune evasion proteins to expression of a large number of extracellular toxins including the α-hemolysin encoded by *hla* [2]. Within RNAIII itself a toxin, the δ toxin, is also encoded [3]. *S. aureus* QS has been demonstrated to be important for virulence in several animal models of acute infection, including infective endocarditis, skin and soft tissue infections and septic arthritis [4–6]. Yet *S. aureus* QS defective mutants are commonly found in clinical isolates and they are associated with a wide range of infections such as persistent bacteremia; infections of the lungs of cystic fibrosis patients and with higher mortality in general [7–11]. Also, in the laboratory they arise spontaneously at high frequencies [12–14]. Recently we showed that exposure to sub-lethal antibiotic concentrations increases the fitness cost of the *agr* system by inducing RNAIII expression levels [15]. The fitness advantage of the *agr* mutant over the wild type (WT) strain could not be related to any differences in exponential growth rate and was only detected in competition between the two strains [15]. These observations prompted us to examine the hypothesis that the apparent fitness advantage of the *agr* mutant cells compared to the WT can be explained by differences in viability.

## Main text

### Methods

#### Bacterial strains and growth conditions

*Staphylococcus aureus* strains (Additional file 1: Table S1) were grown in Tryptic Soy Broth (TSB) containing 2.5 g/l glucose (Sigma-Aldrich) or in Bacto™ Tryptic Soy Broth without glucose, Benton Dickson (BD286220). Blood agar plates contained 1.5% Agar (Difco) and 5% calf’s blood. Antibiotics were added in the following concentrations: tetracycline 2 µg/ml; chloramphenicol 10 µg/ml; erythromycin 10 µg/ml (Sigma-Aldrich). Mutations and plasmids were transferred by transduction using phage φ11 [16, 17]. Transposon mutant clones were obtained from the Network on Antimicrobial Resistance in *S. aureus* (NARSA). *agr* activity was assayed on blood agar plates and hemolysis was scored for approximately 100 colonies.

For *hld* overexpression, pTX_Δ_-RNAIII, was equipped with an *E. coli* replication origin and ampicillin selection marker from pUC19 using primers pUC-1 and pUC-2 (Additional file 1: Table S2) through *Sma*I/*Sac*I restriction sites yielding plasmid pTX_Δ_-RNAIII-pucori. The *hld* coding sequence was amplified using primers Hld1 and Hld2 (Additional file 1: Table S2) followed by the substitution of RNAIII for *hld* using restriction sites *Bam*HI/*Mlu*I yielding plasmid pTX_Δ_-Hld-pucori.

#### Competition experiment

Five independent cultures of Δ*agrA* plus WT cells were grown in TSB with or without glucose for 100 generations (10 passages) with 1 × 10^6^ bacteria transferred daily into fresh growth medium. The ratio of the tetracycline-resistant Δ*agrA* mutant cells to WT cells was determined on TSB agar plates with and without tetracycline after 30, 50 and after 100 generations of growth.

#### Live/dead staining

Staining was performed with thiazol orange (TO) (Sigma-Aldrich) at 0.168 μM and propidium iodide (PI) (Sigma-Aldrich) at 8 μM, staining 10^6^ cells/ml for 15 min under dark conditions at room temperature. The flow cytometry data was recorded with a BD Biosciences Accuri C6 flow cytometer counting 50,000 cells at a flow rate of 35 μl/min and with a core size of 16 μm. Stained cells were excited with a 488 nm argon laser and emission was detected with the FL1 emission filter at 533/30 nm using FL1 photomultiplier tub and in FL-3 emission filter at 670 nm using FL3 photomultiplier tub.

#### Quantitation of RNAIII expression and eDNA levels by real-time qPCR

The SV RNeasy Mini Kit (Qiagen) was used for isolation of RNA; cDNA RT kit (Applied Biosystems) for cDNA synthesis (using an RNase inhibitor, Applied Biosystems) and the FastStart Essential DNA Green Master (Roche) for qPCR in a Lightcycler 96 (Roche) with the primers listed in Additional file 1: Table S2. Data analysis was performed in the LightCycler Application Software, version 1.1 (Roche). Extracellular, chromosomal DNA (eDNA) was quantified by qPCR amplifying the *ileS* sequence directly on 100-fold diluted and heat-treated culture supernatants. The eDNA concentration in the supernatants were subsequently calculated from a standard curve of purified genomic DNA and normalized using an exogenous DNA spike which was added to all samples. The DNA spike was PCR product of the GFP gene and 10,000 copies were added to each qPCR reaction (primers used are listed in Additional file 1: Table S2.

#### Growth potential of lysed cells

*Staphylococcus aureus* cells in TSB were lysed by applying bead-beating (FastPrep^®^-24, MP Biomedicals). Increasing concentrations of lysate were added to TSB diluted 1:10 with water, inoculated with *S. aureus* Newman overnight cultures (diluted 1:100) and incubated in a Bioscreen C reader (Thermo Labsystems) at 37 °C for 24 h. Technical quadruplicates and biological triplicates were included for each condition.

### Results

#### Cell death is reduced in *agrA* and RNAIII mutant cells

Initially we assessed the frequency with which *agr* negative cells arise in strain Newman by passaging five individual WT colonies in serial batch cultures and determining the frequency of hemolysin negative mutants on blood agar plates. Although only a fraction of *agr* mutations will eliminate hemolysis it has previously been used as an indicator of *agr* activity [14]. Non-hemolytic colonies appeared on day 7 and by day 19 haemolytic colonies could only be detected in one lineage (Additional file 1: Figure S1). Thus, in line with findings for other *S. aureus* strains [14], *agr* mutants readily arise in cultures of stain Newman during serial passage.

**Fig. 1:**
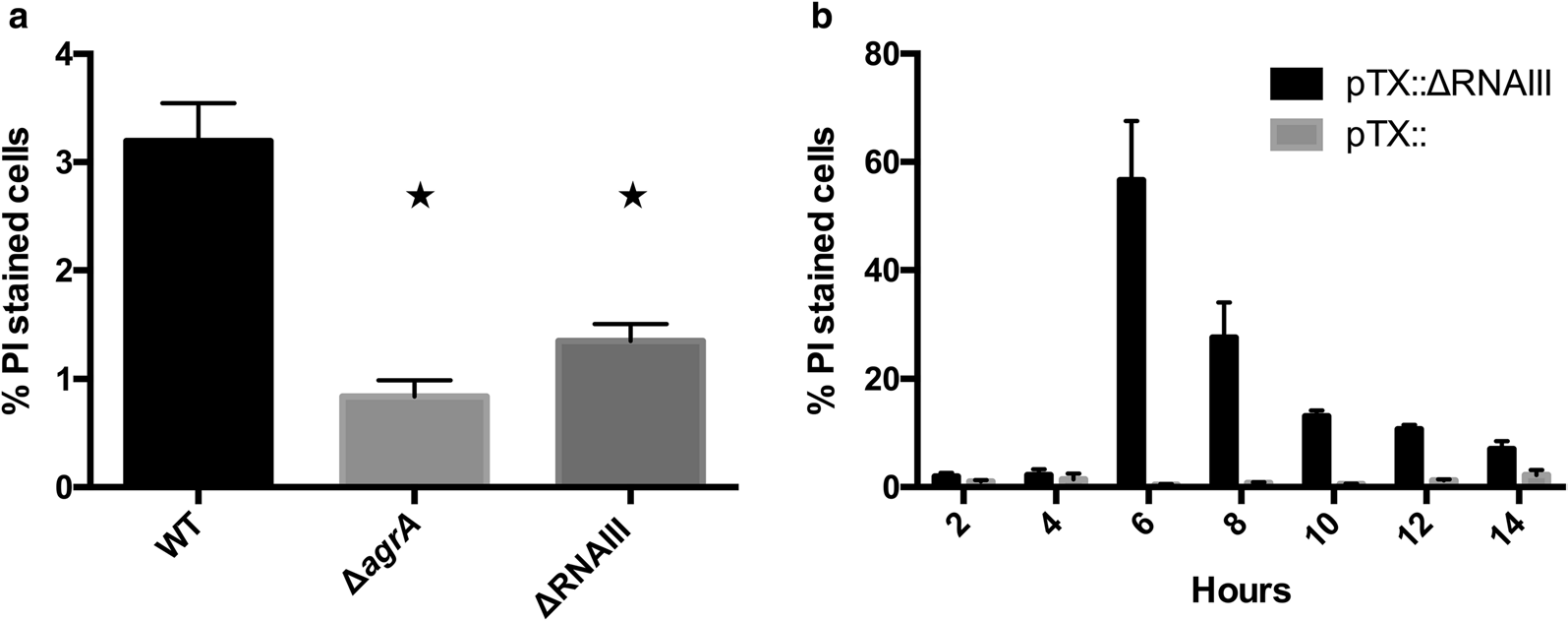
*agr* and RNAIII influence cell viability. **a** Cultures of WT, Δ*agrA* and ΔRNAIII mutant were stained with PI and TO and the percentage of dead cells determined by flowcytometry (An asterix indicates a *p*-value of < 0.05 when compared to WT by Student’s T-test). **b** The percentage of dead cells was determined by staining and flowcytometry at various time points after inoculation of WT cells carrying either vector (pTX_Δ_) or the RNA overproducing plasmid (pTX_Δ_RNAIII). Bars represent the mean and the standard deviation from biological triplicates

Since the selection for *agr* negative cells during the serial passage cannot readily be explained by differences in growth rate and is primarily detected in competition with WT cells [14, 15] we examined the viability of *agr* positive and negative cells. Stationary phase cultures of Newman WT, Δ*agrA* and ΔRNAIII mutant derivatives were live/dead stained with propidium iodine (PI) and thiazole orange (TO) and analysed by flow cytometry. Interestingly, we observed a significantly higher fraction (*p* < 0.05) of dead WT cells compared to Δ*agrA* or ΔRNAIII mutant cells (Fig. 1a) indicating that a fraction of *agr* positive cells loose viability by a process not taking place in Δ*agrA* or ΔRNAIII mutant cells.

#### RNAIII overexpression induces lysis

To address if RNAIII may be the factor reducing the viability in *agr* positive cells, we examined populations of WT cells containing an empty vector, pTX_Δ_, or a plasmid constitutively expressing RNAIII, pTX_Δ_RNAIII. Cultures of both strains were inoculated to a cell density of 5 × 10^6^ colony forming units per ml, growth as well as the live/dead ratio was continuously monitored with flow cytometry. We observed that with the pTX_Δ_RNAIII construct, substantial cell death occurred after 6 h of growth with more than 60% of the population stained as dead cells (Fig. 1b) whereas few dead cells were observed in cells carrying the empty vector, pTX_Δ_. At this timepoint the cells carrying pTX_Δ_RNAIII overproduced RNAIII tenfold above the level in cells carrying the vector (Additional file 1: Figure S2). Upon progression into stationary growth phase, the number of cells stained as dead decreased with RNAIII overproduction (Fig. 1b) indicating that dead cells lyse and consequently are not detected in the flow cytometer. This observation was confirmed by monitoring the release of chromosomal DNA by qPCR. After 7 h of growth, extracellular DNA (eDNA) could only be observed in the supernatant of cells with RNAIII overproduction and it appeared at a concentration of 13.9 µg/ml (± 4.0) eDNA detected, corresponding to the DNA content of 5 × 10^9^ *S. aureus* cells/ml. To assess whether the pronounced cell death observed with RNAIII overexpression was due to overproduction of the δ-toxin encoded by *hld* within the RNAIII transcript, we overproduced δ-toxin from a construct not expressing RNAIII. To this end we removed the entire RNAIII-encoding region and inserted *hld* with the same Shine Dalgarno sequence as on the native transcript. With this plasmid cell death was reduced to only 10% of that seen with the RNAIII-overproducing plasmid showing that the effect is mediated via RNAIII and not the δ-toxin.

#### Bacterial lysis releases resources supporting growth

Since dead cells potentially are cannibalized we examined whether lysed *S. aureus* cells could support growth. For this purpose, we inoculated WT cells in dilute TSB broth (0.1 × TSB) supplemented with varying amounts of staphylococcal lysate obtained from mechanical disruption of *S. aureus* WT cells and observed that increasing amounts of lysate stimulates growth as observed by a higher final optical cell density (Additional file 1: Figure S3).

*Staphylococcus aureus* is known to encode a number of autolysins and we speculated that one of these might be responsible for the cell death. However, upon transduction of mutations in *lrgB*, *cidA*, *lrgA*, *lytM*, *tagX*, a lysM domain protein (NE1640) and an autolysin (NE1948) from the NARSA transposon library into Newman + pTX_Δ_RNAIII none of the mutations altered the lysis phenotype elicited by RNAIII overproduction (data not shown) indicating that the examined gene products are not responsible for the RNAIII mediated cell death.

#### Modulation of RNAIII expression eliminates the competitive advantage of *agr* negative cells

**Fig. 2:**
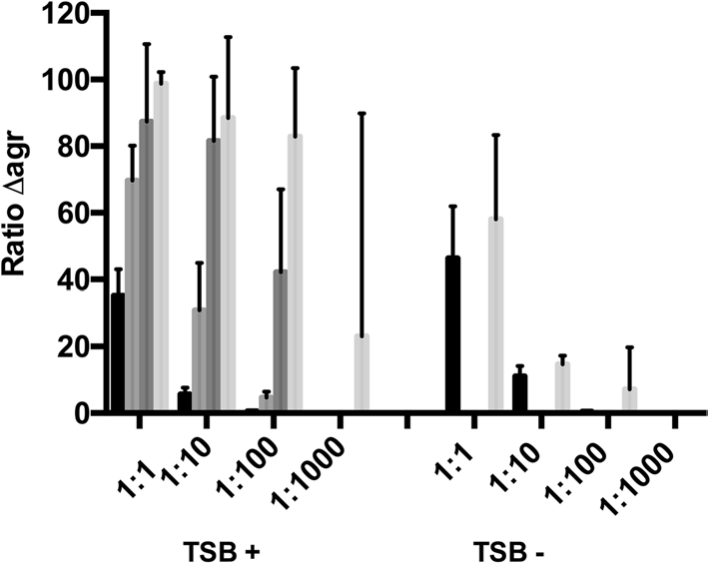
Competition between WT and Δ*agrA* cells. WT and Δ*agrA* mutant cells were co-cultivated in ratios 1:1; 1:10; 1:100 or 1:1000 of WT to Δ*agrA* the presence (TSB+) or absence (TSB−) of glucose for either 30 (grey), 50 (dark grey) or 100 (light grey) generations and the ratio of Δ*agrA* to WT cells was determined based on agar plates with and without tetracycline compared to the inoculum (black). The bars represent the mean and standard deviation obtained from five independent co-cultures

To determine if RNAIII expression levels influences the competition between WT and *agr* mutant cells we competed Δ*agrA* with WT cells and observed that Δ*agrA* cells quickly outcompeted the WT in regular TSB medium (Fig. 2) while this was not the case in TSB lacking glucose where RNAIII expression has been demonstrated to be reduced [18] (Fig. 2). These data suggest that the competitive advantage of being *agr* negative is associated with less RNAIII expression and less lysis of cells.

### Discussion

Here we show that a small fraction of a WT *S. aureus* population undergoes cell death and that this does not take place in mutant cells lacking the *agr* QS system. Since both *agr* positive and negative cells multiply on lysed staphylococcal cells, *agr* negative cells have an advantage over WT cells. We propose that this phenomenon contributes to the frequent manifestation of *agr* mutant cells both in vivo and in vitro during serial passage [14]. Our results agree with previous findings that the apparent fitness advantage of *agr* negative cells is particularly evident in competition assays [15]. Currently we do not know the molecular details of the killing process nor the mechanism behind the stochasticity by which it occurs. However, it has been noted that *agr* mutant cells are less prone to Triton X-100 mediated lysis and are more resistant to lysis by Penicillin G compared to wild type cells [19] indicating that there is an overall basic physiological difference between *agr* positive and negative cells.

In *P. aeruginosa* QS has also been linked with decreased viability as a mutant lacking the *las* QS system resists autolysis at high cell densities resulting in about tenfold increase in *lasR* mutant-to-wild-type ratio in mixed cultures [20]. Interestingly QS negative cells of both *S. aureus* and *P. aeruginosa* appear under chronic infections experiencing prolonged antibiotic exposure [20]. In *S. aureus*, some antibiotics are known to increase expression of RNAIII [9, 21]. We speculate that this induction may lead to increased cell death in WT populations of cells and enhanced the appearance of *agr* mutant cells.

The biological impact of differential death of *agr* positive cells remain obscure. It may contribute to biofilm formation via the DNA released [22] but it may also serve the purpose of establishing mixed populations of *agr* positive and negative cells. As the fraction of *agr* negative cells increases, the induction of *agr* expression in WT cells will decrease and consequently also the RNAIII mediated lethality. In *Salmonella enterica* serovar Typhimurium it has been shown that expression of a phenotypically avirulent subpopulation promotes the evolutionary stability of virulence (35) and similar cooperation may take place in *S. aureus*.

## Limitations

While the phenomenon reported in our study is observed for several strains of *S. aureus* we do not know the extent to which cell death in stationary phase occurs in clinical strains.

## Additional file

**Additional file 1: Figure S1.** Hemolysis negative cells arise in *S. aureus* Newman. Five parallel cultures of WT were passaged in TSB for 24 days. Every second day suitable dilutions were plated out on TSB agar with 5 % calf’s blood and each colony was scored for hemolysis by comparing to WT freshly inoculated from the freeze stock. Zones of hemolysis smaller than ~0,5 mm were scored as hemolysis negative. **Figure S2.** RNAIII overexpression with pTX_Δ_RNAIII. RT-qPCR was used to measure expression of RNAIII in *S. aureus* Newman carrying vector (pTX_Δ_) or RNAIII overproducing plasmid (pTX_Δ_RNAIII) after 6 hours of growth in TSB. Data represent three biological replicates and are shown as mean ratios normalized to a run calibrator of genomic DNA. Error bars represent the standard deviation. **Figure S3.** Lysed bacteria supports growth. WT cells were grown in diluted 0.1xTSB supplemented with increasing amounts of bacterial lysate, and growth was measured in a Bioscreen at OD_600_. The experiment was performed with biological triplicates for each condition and the data represent the mean OD_600_ and standard deviation. **Table S1.** Strains and plasmids used in this study. **Table S2.** Oligonucleotides used in this study.

## Abbreviations

QS: quorum sensing
TSB: tryptic soy broth
eDNA: extracellular chromosomal DNA
