# Supplementary material for: The agr quorum sensing system in Staphylococcus aureus cells mediates death of sub-population
Source: BMC Res Notes. 2018 Jul 24;11:503. doi: 10.1186/s13104-018-3600-6 (PMC6057012; doi:10.1186/s13104-018-3600-6)
Supplement: Supplementary file 1 — Additional file 1: Figure S1. Hemolysis negative cells arise in S. aureus Newman. Five parallel cultures of WT were passaged in TSB for 24 days. Every second day suitable dilutions were plated out on TSB agar with 5 % calf’s blood and each colony was scored for hemolysis by comparing to WT freshly inoculated from the freeze stock. Zones of hemolysis smaller than ~0,5 mm were scored as hemolysis negative. Figure S2. RNAIII overexpression with pTXΔRNAIII. RT-qPCR was used to measure expression of RNAIII in S. aureus Newman carrying vector (pTXΔ) or RNAIII overproducing plasmid (pTXΔRNAIII) after 6 hours of growth in TSB. Data represent three biological replicates and are shown as mean ratios normalized to a run calibrator of genomic DNA. Error bars represent the standard deviation. Figure S3. Lysed bacteria supports growth. WT cells were grown in diluted 0.1xTSB supplemented with increasing amounts of bacterial lysate, and growth was measured in a Bioscreen at OD600. The experiment was performed with biological triplicates for each condition and the data represent the mean OD600 and standard deviation. Table S1. Strains and plasmids used in this study. Table S2. Oligonucleotides used in this study. [file 13104_2018_3600_MOESM1_ESM.docx]

**The *agr* quorum sensing system in *Staphylococcus aureus* cells mediates death of sub-population**

**Wilhelm Paulander, Anders Nissen Varming, Martin Saxtorph Bojer, Cathrine Friberg, Kristoffer Bæk, Hanne Ingmer^*^**

**Additional file containing Additional figures S1-S3 and Additional tables S1 and S2. Figure S1**


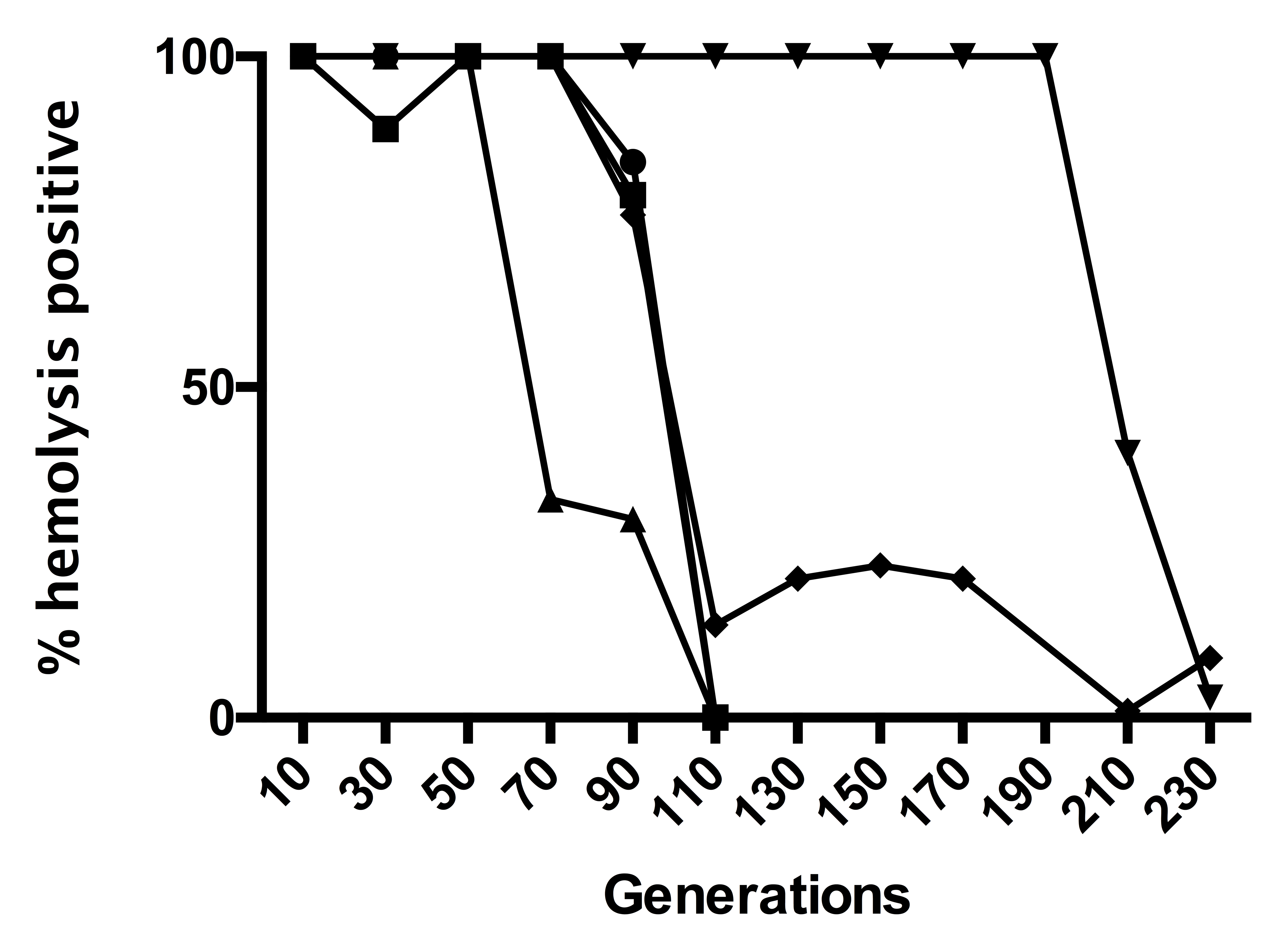


**Figure S1. Hemolysis negative cells arise in *S. aureus* Newman.** Five parallel cultures of WT were passaged in TSB for 24 days. Every second day suitable dilutions were plated out on TSB agar with 5 % calf’s blood and each colony was scored for hemolysis by comparing to WT freshly inoculated from the freeze stock. Zones of hemolysis smaller than ~0,5 mm were scored as hemolysis negative.

**Figure S2**

**
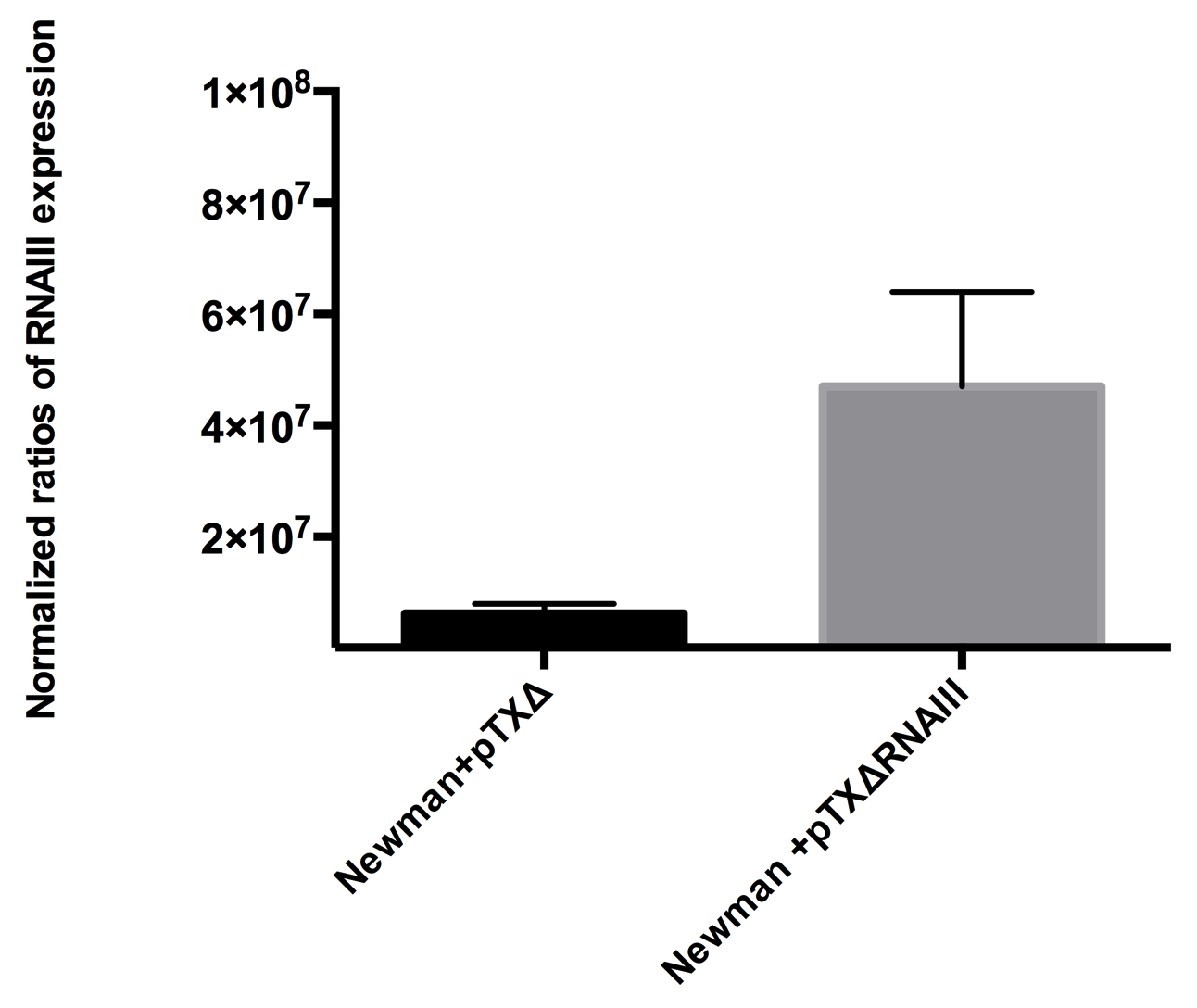
**

**Figure S2. RNAIII overexpression with pTX_Δ_RNAIII.** RT-qPCR was used to measure expression of RNAIII in *S. aureus* Newman carrying vector (pTX_Δ_) or RNAIII overproducing plasmid (pTX_Δ_RNAIII) after 6 hours of growth in TSB. Data represent three biological replicates and are shown as mean ratios normalized to a run calibrator of genomic DNA. Error bars represent the standard deviation.

**Figure S3**


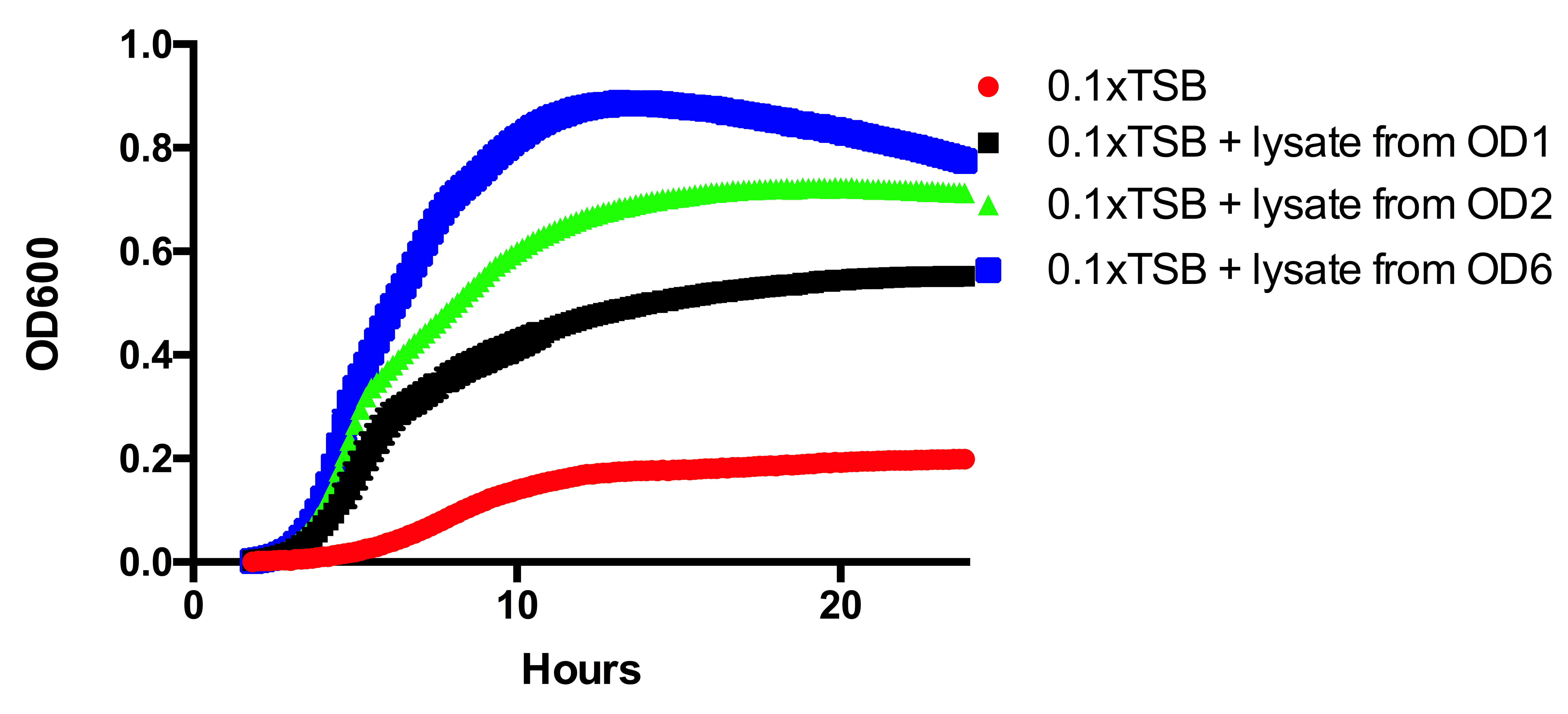


**Figure S3. Lysed bacteria supports growth.** WT cells were grown in diluted 0.1xTSB supplemented with increasing amounts of bacterial lysate, and growth was measured in a Bioscreen at OD_600_. The experiment was performed with biological triplicates for each condition and the data represent the mean OD_600_ and standard deviation.

**Table S1. Strains and plasmids used in this study.**

| Strain / plasmid | Characteristics | Reference |
| --- | --- | --- |
| *S. aureus* Newman | NCTC 8178 wild type Newman | (1) |
| NE 1532 | JE2 with transposon inserted in *agrA* | (2) |
| NE1726 | JE2 with transposon inserted in *lrgB* | (2) |
| NE1692 | JE2 with transposon inserted in *cidA* | (2) |
| NE1438 | JE2 with transposon inserted in *lrgA* | (2) |
| NE641 | JE2 with transposon inserted in *lytM* | (2) |
| NE460 | JE2 with transposon inserted in *atl* | (2) |
| NE1374 | JE2 with transposon inserted in *tagX* | (2) |
| NE1640 | JE2 with transposon inserted in *lysM* domain protein | (2) |
| NE1948 | JE2 with transposon inserted in autolysin gene | (2) |
| WP258 | Newman, transformed with pTX_Δ_RNAIII | (3) |
| WP262 | Newman, transformed with pTX_Δ_ | (3) |
| WP298 | Newman, ΔagrA::Tn551 | (3) |
| WP418 | Newman ΔRNAIII::cat86 | (3) |
| WP424 | WP418, transformed with pTX_Δ_ | (3) |
| WP426 | WP418, transformed with pTX_Δ_RNAIII | (3) |
| AV4 | WP258; *bursa aurealis* transduced into *lrgB* | This study |
| AV5 | WP258; *bursa aurealis* transduced into *cidA* | This study |
| AV6 | WP258; *bursa aurealis* transduced into *lrgA* | This study |
| AV10 + AV11 | WP258; *bursa aurealis* transduced into *lytM* | This study |
| AV22 + AV23 | WP258; *bursa aurealis* transduced into *atl* | This study |
| AV40 | WP258; *bursa aurealis* transduced into *agrB* | This study |
| AV44 | WP258; *bursa aurealis* transduced into *agrC* | This study |
| AV48 | WP258; *bursa aurealis* transduced into *tagB* | This study |
| AV52 | WP258; *bursa aurealis* transduced into *sle1* | This study |
| pTX_Δ_ | tet^R^ | (4) |
| pTX_Δ_RNAIII | Constitutive RNAIII expression; tet^R^ | (4) |
| pDB59 | *agr* P3-yfp_10B_; cm^R^ | (5) |
| pTX_Δ_-RNAIII-pucori | pTX_Δ_-RNAIII with *E. coli* origin | This study |
| pTX_Δ_-Hld-pucori | pTX_Δ_-RNAIII-pucori with RNAIII being substituted with *hld* | This study |

**Table S2. Oligonucleotides used in this study.**

| Oligonucleotide name | Sequence (5’-3’) | Reference |
| --- | --- | --- |
| ileS v.2 F | ACATACAGCACCAGGTCACG | This study |
| ileS v.2 R | CGCCTTCTTCAGTAAATACACC | This study |
| GFP_qPCR v.1.1 F | GATGGAAGCGTTCAACTAGC | This study |
| GFP_qPCR v.1.1 R | GTGGTCTCTCTTTTCGTTGG | This study |
| RNAIII F | GCACTGAGTCCAAGGAAACTAAC | This study |
| RNAIII R | AAGCCATCCCAACTTAATAACC | This study |
| pUC-1 | ATATGTATCCGCTCATGAGAC | This study |
| pUC-2 | GATACAGAGCTCAACGCAGGAAAGAACATG | This study |
| Hld1 | GATACAGGATCCGTGATGGAAAATAGTTGATGAG | This study |
| Hld2 | GATACAACGCGTTTATTTTTTAGTGAATTTGTTCACTG | This study |

**References**

1. **Duthie ES, Lorenz LL**. 1952. Staphylococcal coagulase; mode of action and antigenicity. J Gen Microbiol. **6**:95-107.

2. **Fey PD, Endres JL, Yajjala VK, Widhelm TJ, Boissy RJ, Bose JL, Bayles KW.** 2013. A genetic resource for rapid and comprehensive phenotype screening of nonessential *Staphylococcus aureus* genes. MBio. **4**:e00537-12.

3. **Paulander W, Nissen Varming A, Bæk KT, Haaber J, Frees D, Ingmer H.** 2013. Antibiotic-mediated selection of quorum-sensing-negative *Staphylococcus aureus*. MBio. **3**:e00459-12.

4. **Queck SY, Jameson-Lee M, Villaruz AE, Bach TH, Khan BA, Sturdevant DE,**

**Ricklefs SM, Li M, Otto M**. 2008. RNAIII-independent target gene control by the *agr*

quorum-sensing system: insight into the evolution of virulence regulation in

*Staphylococcus aureus*. Mol Cell. **32**:150-8.

5. **Yarwood JM, Bartels DJ, Volper EM, Greenberg EP.** 2004. Quorum sensing in *Staphylococcus aureus* biofilms. J Bacteriol. **186**:1838-50.
